# Best practice in high-frequency water quality monitoring for improved management and assessment; a novel decision workflow

**DOI:** 10.1007/s10661-025-13795-z

**Published:** 2025-03-04

**Authors:** J. Rozemeijer, P. Jordan, A. Hooijboer, B. Kronvang, M. Glendell, R. Hensley, K. Rinke, M. Stutter, M. Bieroza, R. Turner, P. E. Mellander, P. Thorburn, R. Cassidy, J. Appels, K. Ouwerkerk, M. Rode

**Affiliations:** 1 Department of Subsurface and Groundwater Quality, Deltares, Daltonlaan 600, 3584 BK Utrecht, the Netherlands; 2 Co-Centre for Climate + Biodiversity + Water, School of Geography and Environmental Sciences, Ulster University, Coleraine, BT52 1SA UK; 3 National Institute for Public Health and the Environment, RIVM, P.O. Box 1, 3720 BA Bilthoven, the Netherlands; 4 Institute of Ecoscience, C.F. Møllers Allé 3, Aarhus Univ., DK8000 Aarhus, Denmark; 5 James Hutton Institute, Craigiebuckler, Aberdeen, AB15 8QH UK; 6 National Ecological Observatory Network, Battelle, Boulder, CO USA; 7 Department Lake Research, Helmholtz Centre for Environmental Research - UFZ, Magdeburg, Germany; 8 Department of Soil and Environment, Swedish University of Agricultural Sciences, Box 7014, 75007 Uppsala, Sweden; 9 Reef Catchments Science Partnership, School of the Environment, The University of Queensland, Brisbane, Queensland 4108 Australia; 10 Water Quality and Investigations, Queensland Department of Environment, Science and Innovation, Brisbane, Queensland 4102 Australia; 11 Agricultural Catchments Programme, Department of Environment, Soils and Landuse, TEAGASC, Johnstown Castle, Ireland; 12 CSIRO Agriculture and Food, 306 Carmody Rd, St Lucia, Queensland 4067 Australia; 13 Environment and Marine Science Division, Agri-Food and Biosciences Institute (AFBI), Belfast, Northern Ireland; 14 microLAN BV, Biesbosweg 2, 5145PZ Waalwijk, the Netherlands; 15 Department of Aquatic Ecosystem Analysis and Management, Helmholtz Centre for Environmental Research - UFZ, Magdeburg, Germany; 16 Institute of Environmental Science and Geography, University of Potsdam, 14476 Potsdam, Germany

**Keywords:** Water quality, High-frequency data, Sensors, Monitoring, Decision workflow

## Abstract

The use of high-frequency water quality monitoring has increased over several decades. This has mostly been motivated by curiosity-driven research and has significantly improved our understanding of hydrochemical processes. Despite these scientific successes and the growth in sensor technology, the large-scale uptake of high-frequency water quality monitoring by water managers is hampered by a lack of comprehensive practical guidelines. Low-frequency hydrochemical data are still routinely used to review environmental policies but are prone to missing important event-driven processes. With a changing climate where such event-driven processes are more likely to occur and have a greater impact, the adoption of high-frequency water quality monitoring is becoming more pressing. To prepare regulators and environmental and hydrological agencies for these new challenges, this paper reviews international best practice in high-frequency data provision. As a result, we summarise the added value of high-frequency water quality monitoring, describe international best practices for sensors and analysers in the field, and evaluate the experience with high-frequency data cleaning. We propose a decision workflow that includes considerations of monitoring data needs, sensor choice, maintenance and calibration, and structured data processing. The workflow fills an important knowledge-exchange gap between research and statutory surveillance for future high-frequency water quality sensor uptake by practitioners and agencies.

## Introduction

Routine water quality monitoring for environmental objectives is embedded within statutory obligations in many countries. The monitoring can focus on individual parameters or a combination of chemical, biological, or hydromorphological requirements (e.g. Van Kats et al., 2022). The overarching European Union (EU) Water Framework Directive (WFD) (Carvalho et al., 2019), the United States (US) Clean Water Act (Keiser & Shapiro, 2019), the New Zealand Resource Management Act (Davis & Threlfall, 2006), the Australian Water Act (Skinner & Langford, 2013), and the Great Barrier Reef Water Quality Improvement Plan (Queensland, 2018) are examples with prescriptive water quality surveillance for environmental and ecological objectives. The temporal resolution of this surveillance monitoring varies from country to country and between parameter types (e.g. Jiang et al., 2020; Sundermann et al., 2015) but is typically based on low spatial and temporal resolution. Despite river systems being dynamic on annual, seasonal, and even sub-daily temporal scales, a relatively low temporal resolution approach (e.g. four to twelve grab samples per year, per site in the EU) is practiced over many monitoring stations. With several years of monitoring following standardised field and laboratory procedures, these data are assumed sufficient for policy reviews and decisions under the Driver-Pressure-State-Impact-Response (DPSIR) iterations (e.g. Borja et al., 2006; Lokhande & Tare, 2021; Lu et al., 2019) when combined with other quality elements in statistical frameworks (Kelly et al., 2021). However, recent research questions this assumption, whereby a much higher temporal data resolution is required to detect changes in water quality for policy objectives (McDowell et al., 2024).

This need for high-frequency water quality data can be accommodated by the current growth in in-situ high-frequency water quality monitoring technologies enabling sub-hourly temporal resolutions. One of the earlier developments was for proxy suspended sediment measurements using logged values of calibrated turbidity with solid-state back-scatter optical sensors (Evans et al., 1997; Glendell & Brazier, 2014; Stutter et al., 2017). Since then, further developments have included optical sensors for absorbance and fluorescence (Chapin et al., 2004; Pellerin et al., 2009; Jones et al., 2014; Jones et al., 2019), ion specific electrode (ISE) probes (O’Grady et al., 2022), and wet-chemistry auto-analysers deployed in situ that have capacity to measure macro-nutrients and other major cations, anions, and isotopes (Freyberg et al., 2017; Jordan et al., 2007; Yu et al., 2021). The advances in process understanding in catchment hydrochemistry based on high-frequency water quality monitoring have been subsequently reviewed by Kirchner et al. (2004), Rode et al. (2016a), Pellerin et al., (2016), Burns et al. (2019), and Bieroza et al. (2023). Reviews with a focus on lake and reservoir applications were published by Meinson et al. (2016), and McBride & Rose (2018).

Beyond this curiosity-driven research, high-frequency physico-chemical water quality datasets have been used to identify cases where low-frequency statutory data proved insufficient for adequate policy reviews and decision making in specific settings. For example, Wade et al. (2012) and Halliday et al. (2015) demonstrated that the time of day of sampling physico-chemical parameters (e.g. nutrients, pH, oxygen) showing day-night cycles is critical to ensuring that low-frequency data are not recording false-positive or false-negative assessments when compared against thresholds. Jung et al. (2020) also found a high degree of statistical bias in small (~ 10 km^2^) rural Irish catchments when low-frequency (*n* = 10 year^−1^) phosphorus-P concentration data were related to ecological thresholds, compared with high-frequency (hourly) data. These points were also demonstrated in the UK (Itchen catchment, ~ 400 km^2^) by Fones et al. (2020). In addition, trend analyses based on low-frequency measurements can be biased as was suggested hypothetically by Rozemeijer and Van der Velde (2014) and was observed in a practical case with a changed polder outlet pumping regime (from day to night pumping) by Van der Grift et al. (2016). These examples showed that, depending on the monitoring objective and the variability of the monitored water quality parameters, low-frequency statutory data can lead to misinterpretations of water quality status and trends.

Despite these potential misjudgements and studies showing the potential policy relevant benefits of increased data frequency (e.g. Skeffington et al., 2015), applications of high-frequency water quality monitoring are still dominated by research driven projects and ‘now-casting’ early warning systems using near real-time data for public health purposes (e.g. Gullick et al., 2003; Diehl et al., 2006; Valdivia-Garcia et al., 2016; Burnet et al., 2019). There are fewer examples of changes to the way national water quality datasets are collected for DPSIR frameworks due to evident cost constraints. To aid knowledge exchange, the move from low-frequency grab sampling strategies to those that increase data coverage to near real-time for statutory water quality monitoring in river catchments requires a decision workflow. Vilmin et al. (2018), for example, propose a smarter way of optimising WFD physico-chemical sampling in rivers as a step up from low-frequency data. The approach considers location and applies spatio-temporal interpolators to consider placement of automated and/or seasonal monitoring sites. Using the same reasoning, Jordan and Cassidy (2022) proposed an Options Matrix for considering different forms of physico-chemical sampling approaches from grab, passive, automated water sampling, and in situ using sensors and analysers.

Nevertheless, there are examples where national or state-wide investments in fully automated water quality data capture at sub-daily or hourly scales is linked directly to water policy reviews. In Ireland, for example, six monitoring stations in agricultural catchments monitor P fractions, nitrate, turbidity, and dissolved organic matter synchronously with hydrometeorological parameters, to support reviews on the EU Nitrates Directive (Mellander et al., 2022). In England and Wales, the Environment Agency operates the Environmental Sensor Network of over two hundred sites using a combination of mostly solid-state sensor arrays (YSI & Loewenthal, 2008). In the US state of Iowa, Jones et al. (2018) reported the use of sixty UV nitrate sensors on rivers draining into the Mississippi to assess the state’s contribution to the Gulf of Mexico nitrate load. In Australia, high-frequency monitoring of nitrate and sediment in streams draining to the Great Barrier Reef is undertaken by the Queensland Government typically with optical absorption in situ probes (Roberts et al., 2023). This network is supplemented with a range of local water quality monitoring projects using similar equipment (Davis et al., 2021; Vilas et al., 2020). There has also been a push to supplement discrete water quality monitoring with real-time monitoring to reduce uncertainty in load calculations and consideration for a framework for automated anomaly detection in real-time water quality data (Leigh et al., 2019a, b).

There is also fast growth in technology, including progress with miniaturised (microfluidic) wet-chemistry analysers, low-cost sensor networks (Mao et al., 2019; Nightingale et al., 2019; Saez et al., 2021), and use of more advanced mobile stations (Meyer et al., 2019). At the same time, there are calls from the research community for the realignment of national water quality monitoring to include high-frequency data for environmental policy objectives (e.g. Vilmin et al., 2018; Jiang et al., 2020; Jennings et al., 2022; McDowell et al., 2024).

Despite all scientific and technological successes described and referenced above, the large-scale uptake of high-resolution water quality monitoring is hampered by a knowledge gap regarding practical aspects of implementing sensors and auto-analysers. The scientific literature does not provide comprehensive guidelines on equipment choice, deployment methods, maintenance, data processing practices, and sensor performance. This knowledge gap often precludes ‘turnkey’ solutions for environmental agencies and others. An efficient uptake of high-frequency monitoring equipment would also benefit from consensus and international standardisation (ISO, NEN) on how to integrate the technology in water quality monitoring networks. These challenges were the starting point for this paper where the literature does not currently provide a compendium of practice guidance. The paper is not, therefore, a systematic review of the literature but rather a best-practice explication for knowledge-exchange amongst scientists and regulators.

With this background, using international experiences, the aim of this study was to provide a decision workflow for high-frequency water quality monitoring applications, including:

1. A consideration of the added value of high-frequency water quality monitoring for practical water quality management (when and where to deploy sensors, for what purposes)
2. A description of field practices and considerations (sensor choice, a robust field installation, adequate maintenance, evaluation)
3. Experiences with data processing and optimisation such as dealing with anomalies and data gaps

While the focus of most examples in this paper is on high-frequency monitoring of nutrients in dynamic streams, the principles in the three objectives are applicable to all those water quality parameters that can be monitored in-situ and at high-frequency in any natural water system (e.g. lakes, reservoirs, coastal waters).

## Added value of high-frequency sensor monitoring

The scientific added value of high-frequency water quality monitoring has been described before in several overview papers (Kirchner et al., 2004; Rode et al., 2016a; Van Geer et al., 2016; Meinson et al., 2016; McBride & Rose, 2018; Burns et al. 2019; Bieroza et al., 2023). Kirchner et al. (2004) compared conventional low-frequency sampling with hearing 1 note every minute or two from a Beethoven Symphony, whereas high-frequency monitoring enables researchers to discover the full symphony of catchment hydrochemical behaviour. A recent overview by Bieroza et al. (2023) provides an up-to-date insight into recent successes of high-resolution water quality monitoring. In this section, we provide a short overview of added values of high-resolution monitoring in order to highlight its relevance and to set the stage for the decision workflow for sensor applications the next section.

### Process understanding and model development

The additional value of high-frequency sensor monitoring compared to low-frequency grab sampling depends primarily on the variability of the measured variable. This variability can be driven by underlying hydrometeorological events (transport) and biogeochemical (turnover) drivers or by quick changes in anthropogenic point or diffuse source inputs (Bieroza et al., 2023; Rode et al., 2016a, b; Van Geer et al., 2016). For example, short-term temporal variations in deep groundwater chemistry are limited which reduces the added value of sensor monitoring. High-frequency solute variation in larger rivers is also damped compared to smaller streams (Hensley et al., 2018). The added value of sensors is much higher in freely draining streams with short residence times. For example, capturing the highly dynamic hydrochemistry associated with rapid hydrological responses in conduit systems (e.g. karst and urban areas) and mountainous regions requires high-frequency water quality monitoring (e.g. Yue et al., 2023).

High-frequency measurements can identify all fine-scale temporal variations in water quality and help to understand the underlying hydrological and biogeochemical processes (Rode et al., 2016a). For example, transport-related concentration fluctuations in streams are generally caused by quick changes in the contribution of flow pathways and contaminant fluxes during hydrological runoff events. Rapid changes of matter fluxes can also be caused by point sources, including combined sewage overflows (CSOs). In addition, event-related water quality fluctuations can be caused by in-stream sourcing when sediment bound compounds are remobilised (e.g. Hallberg et al., 2024; Van der Grift et al., 2016). Depending on the season, several biogeochemical processes in fluvial systems can also induce diurnal nutrient concentration variations.

The availability of high-frequency data also offers a new perspective on process-based model parametrisation. In addition, estimates of instream assimilation and denitrification help to constrain catchment nitrogen delivery and transport models. This also means that sensor networks can help to increase the reliability of new water quality modelling approaches regarding the urgently needed assessment of climate change impacts on nutrient dynamics (Ghaffar et al., 2023; Negri et al., 2024; Yang et al., 2018; Zhou et al., 2022).

It has to be pointed out that the use of high-frequency measurements does not always improve model credibility. If the statistical variability of low- and high-frequency concentration data is not very different, the added value for constraining water quality models can be limited. For example, Phillips et al. (2024) showed that the use of high-frequency phosphorus concentration data did not significantly improve the model performance of the HYPE model compared to the use of monthly measurement data in a case study for southern Ontario. They attributed their findings to similar levels of statistical variability of the low- and high-frequency calibration datasets. Similar findings have been presented by Woodward et al. (2017) for statistical nitrate modelling. In addition, high-frequency monitoring does not always reduce model prediction uncertainty compared to fortnightly measurement frequency (Jiang et al., 2019).

High-frequency data can play an important role in assessing the effects of climate change on water quality as the severity of floods and droughts are expected to increase and event-driven contaminant transport processes are more likely to occur and have a greater impact (Boyacioglu et al., 2012; Baron et al., 2013; Loecke et al., 2017; Bieroza et al., 2020; Warren et al., 2022). However, the responses and mechanisms of river water quality under more frequent and intense hydroclimatic extremes are not well understood (Bieroza et al., 2019; Van Vliet et al., 2023). The use of sensors can help examine chemical concentration–discharge relationships in individual storms and over longer time periods that may also detect evidence of initial changes in response to climate change (Musolff et al., 2021; Fazekas et al., 2021). Moreover, high-frequency data can reveal insightful shifts on drought-induced seasonal patterns of in-stream nutrient turnover (Yang et al., 2023).

For evaluating the added information value of high-frequency data sets, whether with regard to modeling or load estimates, a uniform data thinning experiment with the high-frequency data is recommended (Phillips et al. 2024). The variability of the data can be analysed using simple statistical variability measures or autocorrelation analysis (see also Sun et al., 2022). This could be informative for understanding system variability and could also be the basis for a cost–benefit analysis, where the cost relates to sample acquisition and the benefit lies in the quality of the model or load estimation. Once the model predictions become stationary independent of further data thinning, marginal benefits and costs for the specific monitoring targets can be determined and future monitoring costs can be optimised.

### Compliance testing

The EU WFD requires that the good/moderate (G/M) boundaries for nutrients are compatible with good ecological status for sensitive biological quality elements (BQEs) (European Commission, 2000; Kelly et al., 2021). These nutrient boundaries are derived from established relationships between selected biological indicators under the WFD (macrophytes, benthic invertebrates, chlorophyll *a*, phytobenthos) and the average concentration of nitrogen and phosphorus from traditional monitoring of nutrient concentrations during either the summer season (lakes) or entire year (rivers). Introduction of high-frequency nutrient monitoring will be a more accurate methodology for a statutory control of G/M boundaries in streams and rivers due to the capture of extreme events (Halliday et al., 2015; Wade et al., 2012). Current regulatory compliance testing is, however, still based on conventional, standardised grab sampling monitoring technology. Within this legal framework, water managers may not need the extra temporal resolution for compliance testing. On the contrary, open data policies may force them to report all measured water quality anomalies and to take action to remediate them.

### Load estimates and proxies

Accurate load estimates are a pre-requisite to quantify the pressure on downstream receiving water systems and to evaluate load reduction measures. Traditional water quality monitoring programs rely on the analysis of grab samples that are typically collected at a frequency too low to fully characterise the dynamics of nutrient concentrations and to obtain accurate and unbiased calculations of nutrient loads for different periods of time (month, year) (Scholefield et al., 2005; Leigh et al., 2019a, b). Flow-proportional sampling using auto-samplers triggered by discharge data can produce more reliable load estimates. High-frequency sensor monitoring can capture sub hourly variations in solute concentrations and short load pulses that may be omitted or overlooked by traditional or flow-proportional periodic grab sampling (Horsburgh et al. 2010; McDowell et al., 2024).

**Fig. 1:**
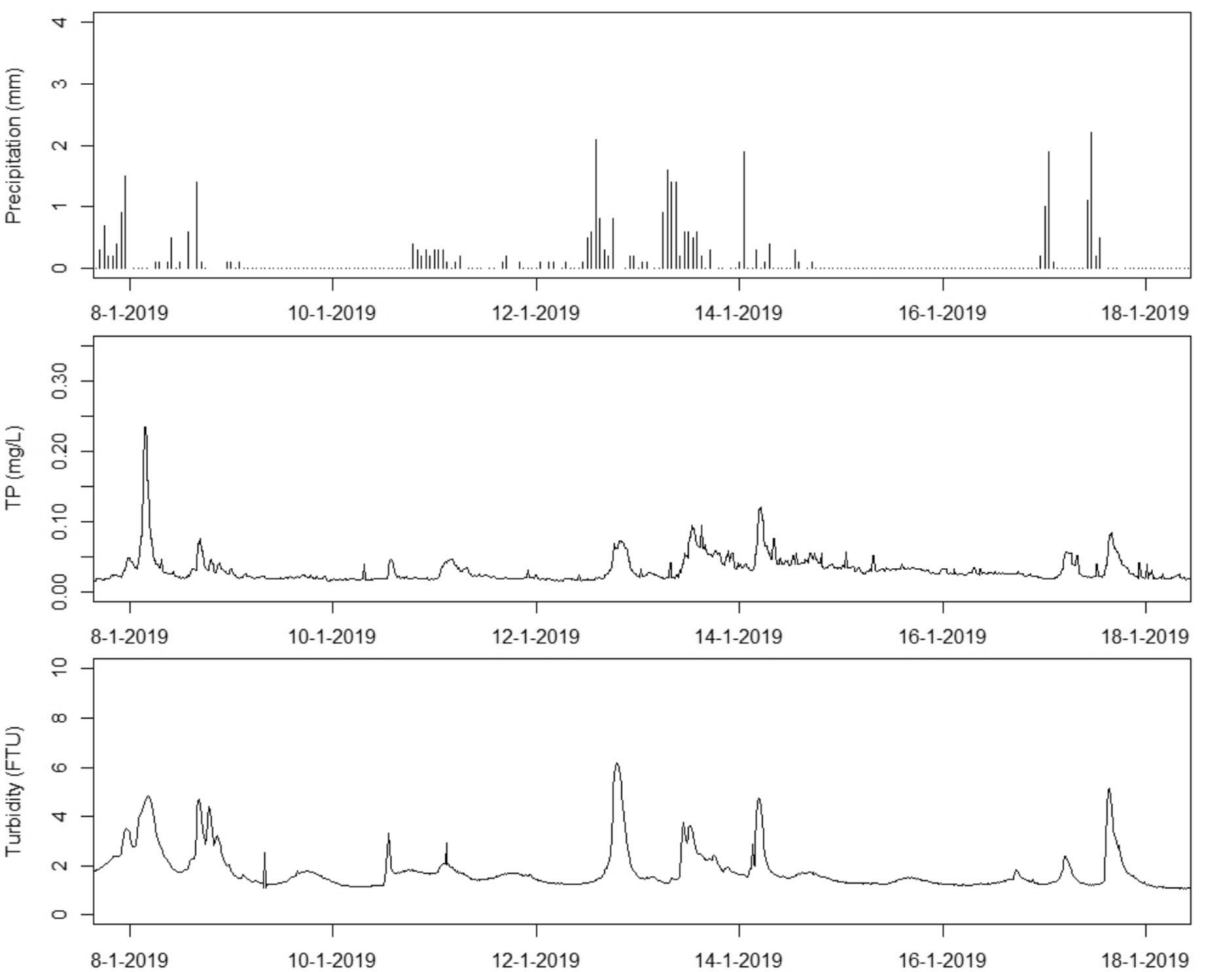
Continuous precipitation, TP, and turbidity data from a farm ditch in East-Netherlands showing the strong correlation between relatively cheap and easy to measure turbidity and relatively expensive TP measurements (adapted from Barcala et al., 2020)

For many compounds direct high-frequency measurements are expensive, but data from relatively cheap and robust sensors for proxies such as electrical conductivity (EC) or turbidity can improve load estimates. For example, using turbidity as a surrogate for suspended sediment concentration is widely accepted because a set of methods is available for generating well defined relationships between those variables (e.g. Glendell & Brazier, 2014; Stutter et al., 2017; Wang & Steinschneider, 2022; Skarbøvik et al., 2023). In addition, turbidity can be used as a proxy for other compounds controlled by their particulate fraction such a total P (TP) or particulate P (PP) (e.g. Barcala et al., 2020; Villa et al., 2019). Figure 1 gives an example of the strong relation between turbidity and TP, which was later captured in a random forest model enabling accurate load estimates (Barcala et al., 2023). Random forest also performed well in predicting high-frequency N and P concentrations from conductivity, dissolved oxygen, turbidity, temperature, pH, chlorophyll, and flow rate in a study by Castrillo and García (2020). Another example is the application of compounds such as fluorescent dissolved organic matter (fDOM) and spectral absorbance coefficient (SAC) as proxies for dissolved organic carbon (DOC) and thereby organic nutrients (Pellerin et al., 2013). The relationships between hydrological variables and solute concentrations can also be captured in statistical or process-based models and applied to reduce the bias in load estimates. For example, Rozemeijer et al. (2010) and Jomaa et al. (2018) significantly improved nitrate (NO_3_) and TP load estimates using high-frequency discharge, groundwater level, and precipitation data. However, the major challenge using surrogates to derive parameters of interest is the large spatial and temporal variability of such relationships, which means that they cannot be directly applied to other streams or catchments without prior calibration.

The use of sensors to measure proxy parameters could help extend the number of monitored sites and improve estimations of nutrient concentrations and loads, especially since lower-cost, robust sensors for long-term field deployment are becoming more readily available (Villa et al., 2019). However, some care is required with proxy data for trend analysis especially for monitoring mitigation actions when processes governing the source or mobilisation of chemicals are decoupled from the processes of transport and association. For example, reduction of fertiliser P at the field scale would not necessarily be associated with a change in turbidity which is itself a proxy for suspended sediment from field or riverbank/bed erosion (Dupas et al., 2015).

### Early warning and operational water management

As summarised in the introduction, high-frequency sensor measurements can be used for ‘now-casting’ to set alert warnings for operational water management purposes. An example is the intensive continuous monitoring in the river Rhine which focuses on early warning for downstream drinking water stations (Diehl et al., 2006). Alerts for sewage or industrial spills of contaminants due to overflows or accidents can be based either on direct measurements or proxies for different inorganic and organic nutrient forms like organic pollutions. Whenever extreme values of a given state variable are reached, i.e. a rare event occurs, a high measurement frequency is required to capture the extreme conditions quantitatively (see for example the river Odra fish kill disaster; Sługocki & Czerniawski, 2023). This can be important for impact assessments, for instance on biological processes or communities in streams, rivers, lakes and reservoirs during critical situations under heat waves.

High-frequency water quality data monitoring can also be applied for adaptive management of water infrastructure such as reservoir operation for drinking water. An example is the extensive real-time monitoring network at Rappbode Reservoir (Rinke et al., 2013), Germany’s largest drinking water reservoir. This reservoir has a bypass which is used whenever the inflow water quality is insufficient, in this case due to high content of humic substances monitored by UV (Ultraviolet) spectroscopy. The high-frequency monitoring helps to operate the bypass, and the archived time-series data helps to identify an optimal set of operation rules as outlined in Zhan et al. (2022). Similarly, selective water withdrawal is profiting from proper monitoring, for example when specific depth layers with poor water quality are selectively taken out of the reservoir to protect raw water quality intake. The effectiveness of such strategies was demonstrated in the example of metalimnetic algal blooms and their mitigation in a modelling study by Mi et al. (2022).

### Effect evaluation

High-frequency monitoring can assist in more accurate quantification of source contributions, such as the distinction between contributions from point sources, natural background sources, and agricultural sources of nutrients (Yu et al., 2021), as well as contributions from different hydrological pathways (Campbell et al., 2015). Therefore, high-frequency sensor monitoring is also vital when testing the effect of implementing a programme of measures as it can capture trends in, for example, P concentrations during extreme events (high flow) (Campbell et al., 2015). Depending on the specific mitigation action, shallow flow routes are expected to respond first, while deeper groundwater contributions can stay unaffected for decades.

High-frequency water quality monitoring enables a quicker detection of trends and effects of mitigation measures compared to low-frequency grab sampling. Trend detection based on low-frequency water quality measurements can also be biased even with multi-year monthly concentration data (McDowell et al., 2024; Rozemeijer & Van der Velde, 2014; Van der Grift et al., 2016). At a larger scale, the Water Framework Directive (WFD) requires detection of downward trends in contaminant concentrations (‘no deterioration’ or ‘stand-still’).

### Stakeholder engagement

By providing more accurate proof of pollution state or effects of mitigation, high-frequency sensor monitoring is also useful for stakeholder engagement to promote behavioural change. This can be further stimulated by making local real-time water quality data available online. This helps to link land management and weather variability to water quality impact (Davis et al., 2021; Vilas et al., 2020). For example, stakeholder groups in five catchments in the Nordic-Baltic region were asked about their opinion on sensor monitoring in the EU project NORDBALT-ECOSAFE. To the question ‘Do you think that sensors can be used to motivate and inform people in the river basin?’, 20–50% of the 6–16 stakeholders involved in the five catchments answered that many people would check online sensor data. Fifty to 70% of the stakeholders expected that at least some interested people would check online sensor data (NORDBALT-ECOSAFE, 2023).

Literature reports on the role of high-frequency water quality monitoring in stakeholder engagement and awareness raising are relatively scarce. Makris et al. (2023) described a near real-time monitoring system of the β-d-glucuronidase activity (as proxy for *E. coli* and other pathogens) to inform citizens about bathing water quality in Breda city in the Netherlands, where recreational use of the urban waters is actively promoted. Another example is the 1622WQ platform visualising real-time nitrate concentrations in Queensland (Australia) to increase farmer awareness of the impact of agriculture on water quality (Vilas et al., 2020).

## Decision workflow for sensor applications

**Fig. 2:**
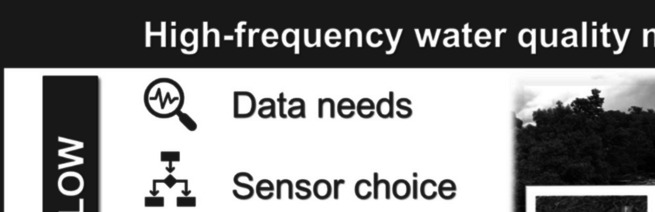
Decision workflow for the implementation of water quality sensors by water quality authorities

To fill a knowledge-exchange gap in water quality sensor use, Fig. 2 presents a decision workflow or ‘cookbook’ for environmental agencies and catchment managers that wish to enter the new era of high-frequency sensor monitoring. The schedule informs the step-by-step choices that are to be made and will further be clarified in this section (data needs, sensor choice, installation, maintenance) and in the next section (data processing).

### Data needs

A first very important step in any monitoring network design following the ‘monitoring cycle’ (MacDonald, 1994; Timmerman et al., 2000) is to consider the general monitoring objectives and data requirements. Specifically for sensor applications, this involves questions such as the following: (1) what is the added value of utilising high-frequency sensor measurements? (2) at which spatial scales is high-frequency sensor monitoring most likely to add value beyond traditional monitoring? and (3) what are the costs compared to traditional grab measurements?

The potential added value of high-frequency water quality monitoring for water quality managers (as outlined in the previous section) can be made more specific within the framework of the monitoring cycle in which the data requirements follow from the monitoring objectives. The added value also depends on the variability of the solute(s) to be monitored. In general, systems with greater temporal variability in solute concentrations require higher frequency sampling for accurate status, load, and trend assessments. Therefore, the advantage of high-frequency monitoring is significant in dynamic, regional catchments compared to large water bodies with long residence times.

**Fig. 3:**
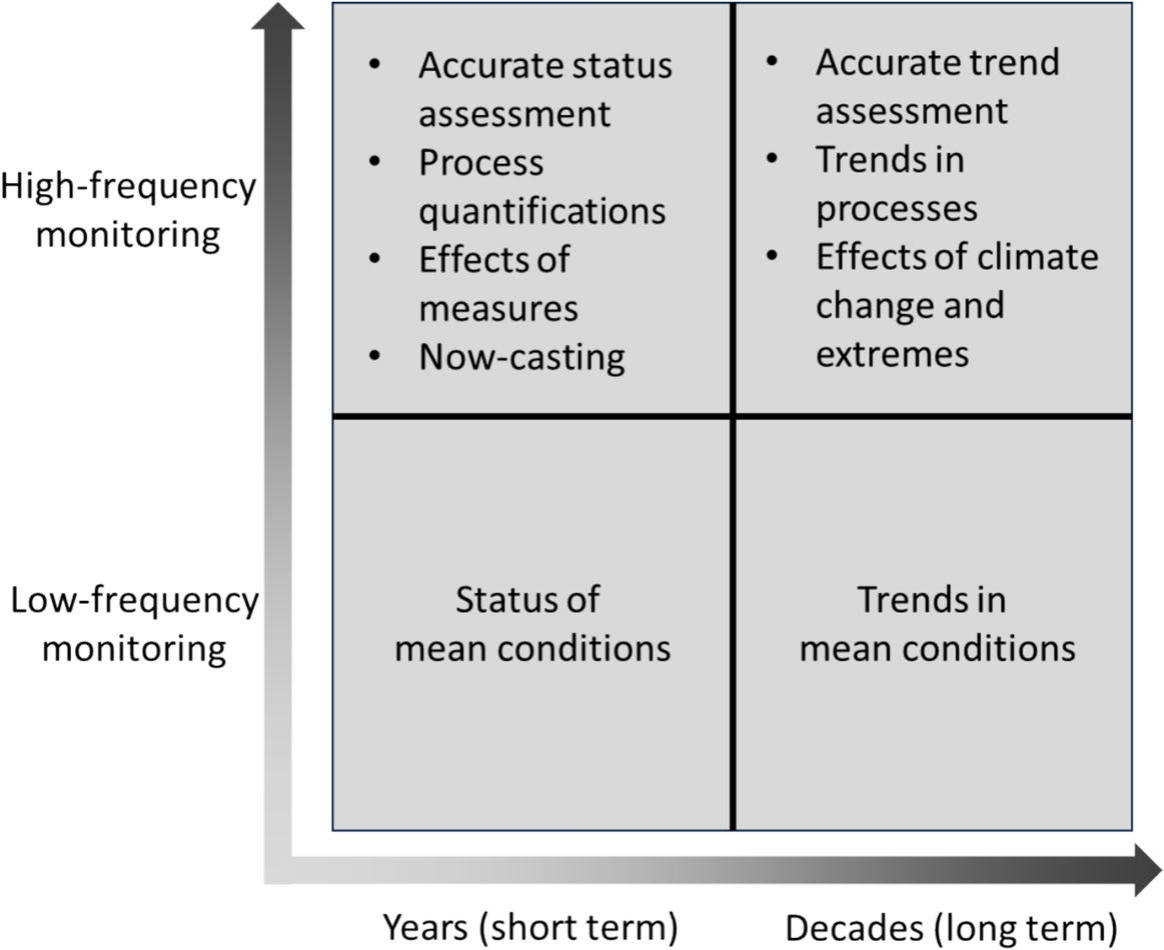
Overview of the added values of utilising high-frequency sensors compared to traditional low-frequency grab sampling for short- and long-term applications

Figure 3 provides a scheme with the general benefits of short-term (1–5 years) and long-term (> 5 years) high-frequency water quality monitoring in a dynamic catchment. Short-term monitoring studies using conventional low-frequency (e.g. monthly) sampling (the lower-left quadrant in Fig. 3) will in many cases only enable uncertain status assessments of the mean conditions (see e.g. McDowell et al., 2024). Using high-frequency monitoring for a short period (upper-left quadrant in Fig. 3) can deliver a more certain status assessment, but can also provide process identification and quantification, evaluation of effects of measures, and now-casting applications for operational water quality management. In long-term monitoring studies, conventional monitoring (lower-right quadrant) can deliver trend assessments for mean to low flow conditions with a relatively high uncertainty, although large-scale networks can achieve acceptable reliability by including large numbers of monitoring locations (space-for-time compromise). However, long-term high-frequency monitoring (upper-right quadrant) can deliver accurate trend assessments, changes in processes, evaluation of effectiveness of measures, and insights in, for example, effects of climate change and climate extremes on water quality.

The costs of applying high-frequency water quality monitoring depend on the parameter and the type of equipment chosen, but some guidance about what to include in cost estimates from current applications is given in Table 1. Despite the considerable costs of high-frequency water quality monitoring, the benefits towards water quality management and societies are expected to exceed the costs in many application cases. However, general cost-benefits analyses are hard to make because both the costs and the benefits are case-specific. The benefits of high-frequency monitoring (as outlined in the section ‘Added value of high-frequency sensor monitoring’) are also hard to quantify, as is also the case for conventional water quality monitoring. The gained knowledge on transport processes, nutrient loads, and mitigation effects feeds into all modeling, monitoring, and mitigation aspects of water quality management. This prevents costs of implementing the wrong mitigation measures at the wrong locations. Early warning towards drinking water intake sites based on high-frequency monitoring (Diehl et al., 2006) prevents costs for extra water purification. Increasing stakeholder involvement through real-time water quality data may help achieving water quality policy targets more effectively. Seifert-Dähnn et al. (2021) performed a cost-benefits analysis of high-frequency water quality monitoring for lakes. They reported the prevention of human health impacts and reputational damages as the most important benefits, although they did not always outweigh installation and operation costs (Seifert-Dähnn et al., 2021). Estimating and evaluating the added value and the (cost-)effectiveness of high-frequency water quality monitoring beyond conventional monitoring is recommended for all applications.

**Table 1:** Overview of the different cost elements to be considered when applying high-frequency monitoring equipment for nutrients (costs estimated by the author group)

Cost element	Details and suggestions for costing and applications	Typical costs (in 2024)
Cost of acquiring a sensor	One time cost depending on sensor type	UV sensor^a^ (NO_3_): 10–20 k€ ISE^b^ (NO_3_): 5–10 k€ Wet chemical^c^ (TP): 50–100 k€
Depreciation period	Number of years until replacement	UV sensor^a^ (Nitrate): 5–10 years ISE^b^ (Nitrate): 0.5–5 years Wet chemical^c^: 10–15 years
Cost for establishing a high-frequency water quality station	Typical installation costs depend on deployment method but involves typically: power supply; material for installation such as e.g. construction costs for sensor deployment directly in the stream or in a cabin of some kind. Wet chemical sensors will always have to be installed in a cabin	Power supply: 3–6 k€ UV sensor^a^ in-stream: 2–3 k€ ISE^b^ in-stream: 1–2 k€ Cabin: 10–20 k€ Dataloggers and data transfer (telemetry): 2–5 kEUR
Annual operating expenses	Annual cost for power, wipers, ISE-tips, membranes, chemicals, etc	UV sensor^a^: < 0.5 k€ per year ISE^b^: 0.5–2 k€ per year Wet chemical^c^: 2–5 k€ per year
Annual cost for maintenance and calibration	Maintenance depends heavily on sensor type but might include regular manual cleaning of sensors (e.g. monthly intervals), calibration of sensor (in-situ zero point offset test), change of membranes, supply of new chemicals and water samples analysed at laboratory (e.g. monthly) the latter being normal part of grab sampling programs	UV sensor^a^: 2–5 k€ per year ISE^b^: 10–20 k€ per year Wet chemical^c^: 3–6 k€ per year
Costs for establishing database for sensor data and making data available	Might utilise existing database to be further developed to include high-frequency data (e.g. data capture at a frequency between 1–30 min)	10–20 k€
Cost for cleaning of sensor data (anomaly detection and gap filling)	Might use existing publicly available software for cleaning and gap filling	10–20 k€
Cost for data interpretation and/or decision support systems	Depends on the complexity of the hydrochemistry at the field site and collected data, the use of external data (e.g. weather data) supporting the interpretation or decision making, and the level of automation	10–100 k€

^a^UV sensors are based on ultraviolet (UV) light absorption

^b^ISEs are ion selective electrodes

^c^Wet chemical are auto-analysers using reagents

### Sensor choice

In general, the sensor choice (second step in the workflow in Fig. 2) involves considering different types and brands and how their characteristics fit with the monitoring requirements and available resources. The interest in high-frequency monitoring has increased in recent years and choices must be made in both sensor type (measuring method) and sensor brand. For nutrients, four general sensor types are available: ultraviolet (UV) sensors, ion selective electrodes (ISEs), wet-chemical auto-analysers, and microfluidic sensors (Table 2). Although auto-analysers and microfluidic sensors have a laboratory-like sample treatment and analytical procedure, we include them here as they are applied in situ for high-frequency water quality monitoring. As indicated in Table 2, TP and TN (total nitrogen) can only be analysed with a wet-chemistry auto-analyser including particulate matter destruction by adding acids and/or heating.

**Table 2:** Characteristics of sensor types for nutrients

Characteristics	UV	ISE	Auto-analyser	Microfluidic
Nutrients	Nitrate	Nitrate, ammonium	Nitrate, ammonium, total nitrogen, phosphate, total phosphorus	Nitrate, ammonium, phosphate
Price	Moderate	Cheap	Expensive	Moderate
Size	Moderate	Small	Large	Moderate
Stability	Moderate	Unstable	Stable	Stable
Accuracy	High	Moderate	High	High
Power consumption	Moderate	Low	High	Moderate
Detection range	Large	Small	Large	Large
Maintenance efforts^a^	Low	High	Moderate	Moderate

^a^Maintenance efforts depend on the sampling conditions, e.g. in highly turbid and eutrophic streams more fouling (formation of deposits caused by biological, chemical and/or physical processes) on the equipment will occur

The sensor choice follows from the desired accuracy, the field situation, and available resources (finance, power supply, technicians, space, location, maintenance requirements). In general, the ISE has a lower stability and higher maintenance demand compared to the UV and auto-analyser. In addition, ISEs may pick up electronical interference from other nearby sensors leading to inaccuracies and instability. The deployment method is also important; for example, a UV sensor placed directly in a stream will probably be outperformed by a UV sensor in a better protected and controlled bank-side analyser system. ISEs are generally cheaper, smaller, and have a lower power consumption compared to UV sensors and auto-analysers. Auto-analysers require protective housing and have a high power consumption and their application is most realistic on larger, permanent monitoring sites where data quality is important. Auto-analysers often have flushing routines after each measurement and programmed cleaning and calibration routines at e.g. daily intervals. Therefore, lower maintenance frequencies are usually allowed, although the auto-analyser maintenance can be more complex and labour intensive compared to ISE and UV sensor maintenance.

Independent inter-manufacturer sensor tests are rare in the literature and new sensor type releases make them quickly incomplete. Characteristics about performance in sensor documentation usually come from manufacturers’ tests in very stable conditions. In practice, frequent field calibrations are always required as each site has its unique circumstances which may affect sensor performance. Independent user experiences often give a more reliable picture of sensor quality and user friendliness. Sensors clearly differ in quality and construction, which might lead to higher overall maintenance and calibration costs over time. Some experiences with sensor comparisons are summarised in the following paragraphs.

Pellerin et al. (2013) tested four different UV nitrate sensors (HACH Nitratax, Satlantic SUNA, S::CAN spectrolyzer, and TriOS ProPS). Their advice is to consider the concentration ranges for nitrate and matrix elements (like DOC and suspended matter) together with logistical constraints. The UV nitrate sensors from individual manufacturers differed in several important ways that affect their ability to accurately measure in-situ nitrate concentrations in different systems.

Hooijboer et al. (2021) compared seven different nitrate sensors (six UV sensors and one auto-analyser) in the Meuse river water and found that the differences between the sensors are mainly determined by their initial calibration. While the absolute measurement values differed between sensors, the temporal variation is measured equally well. These findings suggest that low-frequency laboratory measurements are still valuable at sensor locations to detect the potential off-set and correct the sensor values.

Beyond nutrients, sensors for turbidity were compared by Rymszewicz et al. (2017) and fluorometers for measuring dissolved organic matter were evaluated by Downing et al. (2012).

### Installation

As a third step in the workflow (Fig. 2), a consideration of installation aspects such as equipment housing and power supply is needed (Table 3). In general, the two main types of configurations used for high-frequency water-quality monitoring locations are bank-side or in-stream. In a bank-side setup, a flow-through monitoring system has a pump that delivers water from the measuring point in the stream to the sensors housed in a shelter (e.g. Wagner et al., 2006). This can be performed by a flow-through cell around the sensor or a reservoir in which the sensors are placed. In in-stream setups, the sensors are placed directly at the measuring point in the aquatic environment (Wagner et al., 2006).

**Table 3:** Summary of sensor installation aspects (translated from Van Herpen et al., 2022 (in Dutch))

Element	Option	Pros	Cons
Powering	Mains power	Reliable also with large power consumption (wiper or compressed air cleaning, pumping, climate control)	Not much availability in remote fields; time and cost for new connections
	Battery + solar panel	Stand alone	Lower power supply in winter, theft-sensitive
	Only battery pack	Works without sun	Not sustainable, replacement efforts
Housing	In situ, no housing	Fairly simple	Fragile
	In situ, perforated tube	Simple protection against litter	More stagnant water, growth of plants around
	Flow-through cell	Controlled circumstances	More energy consumption
	Flow-through reservoir	Controlled circumstances, filtration possible	Large housing needed, more energy consumption
Cleaning	None/by hand	Can be performed very carefully	Labor intensive
	Wiper	Easy to install	May cause damage to lens
	Compressed air	No damage, elegant way of cleaning	More hardware needed, more power consumption
Positioning	Floating	Always the same measuring depth relative to water surface	More complex Installation with floater, may get stuck, not possible in shallow water
	Fixed level	Easier to install, may run dry above water level	Measurements at different depths relative to water surface

The conditions in bank-side setups are more controlled and less sensitive to weather extremes. However, power consumption is higher and pumps and pipes or tubes can clog. A bank-side setup is especially preferable if the availability of water is small, such as in ephemeral drainage ditches. Within the flow-through cell, the sensor can be protected from, for example, drying out, frost, high temperatures and disturbance by wildlife. However, pumping and pump tubing may affect sediment-bound constituents or gases in solution such as oxygen.

In-stream monitoring is easier to install, uses less power, but circumstances are less controlled. There are several ways of installing in-stream monitoring systems. The design of infrastructure used for deploying water quality sensors (e.g. housing, sensor depth (Erwin et al., 2021)) can affect the data quality. Despite this, sensor infrastructure design has not been well discussed in the literature (Hensley et al., 2021). Hensley et al. (2021) found minimal differences between two ways of installing an in-stream monitoring system: one with a monopod inside the stream bed and one hanging from an overhead cable. For an in-stream monitoring system one has the choice between a fixed level or a floating sensor. Floating systems can be useful in systems with varying water levels and can prevent sensors from running dry above the water level. In addition, a floating system may correspond better with conventional grab sampling at fixed depths below the water table. Additional considerations for in-stream monitoring include protecting the sensors from being washed away in high flow events or becoming dry during periods of low flow (both extremes are becoming more frequent with climate change). Fouling on the equipment (formation of deposits caused by biological, chemical, and/or physical processes) can cause drift and necessitates easy access to the sensors for regular cleaning and calibration. Finally, in publicly accessible locations, physical measures to protect sensors from vandalism will need to be considered. In this situation, often installation of notices explaining the purpose of the equipment to the public may be an effective means to reduce this risk.

A bottleneck for sensor installation can be the power consumption of the equipment. Monitoring stations are often situated in remote places in rural landscapes where mains electricity is not always available. For systems using solar or wind power, sufficient battery power should be available even during dark and/or wind-free days. This may limit the application of energy-intensive auto-analysers and bank-side setups where pumping and climate control require additional power.

Following sampling theory (e.g. Wu & Thompson, 2020), the statistical distribution of the measurements (the samples) should correspond with the statistical distribution of the real system (the population). Therefore, the desired minimal water quality monitoring frequency depends on the concentration variability. The concentration variability at new field sites is usually not known beforehand, but high frequency monitoring equipment using, for example, sub-hourly to hourly intervals typically captures the full variability and usually oversamples in time. Depending on the equipment used, it can therefore be useful to optimise the monitoring frequency based on the first high-frequency data collected at a new site. ISE and UV sensors can measure continuously without much higher consumption of energy or other resources compared to lower frequencies. Results from these sensors are typically stored at ten-minute intervals, capturing potential sub-hourly water quality dynamics while not producing too large data files for storage and/or online data transfer. For auto-analysers and microfluidic sensors, the minimal frequency depends on the duration of the analytical procedure. This can involve pretreatment of the sample (filtration, homogenisation, heating), adding and mixing of reagents, and the chemical reaction time. Although sub-hourly frequencies are usually possible, lower frequencies could be considered in order to reduce the amount of resources (reagents, energy, maintenance) needed. In this case, we recommend starting up the monitoring at new sites at the maximum frequency possible. After the first months, the gathered data can be used to evaluate whether a lower frequency could also fulfill the monitoring objectives.

### Maintenance

The crucial fourth step in the workflow (Fig. 2) considers the usually frequent maintenance (cleaning and calibration) needed by high-frequency sensor monitoring systems. Fouling is one of the main causes of inaccuracies of sensor measurements (drift). Required maintenance varies in space and time as it depends on local conditions. For example, iron concentration, redox-situation, salinity of the water, carbonate precipitation, and biological activity can all cause fouling issues. Automatic cleaning systems are an essential requirement as they reduce manual maintenance and increase the accuracy and stability of optical and other electronic sensors. Automatic cleaning for UV sensors can be either performed by a mechanical wiper or with compressed air. Auto-analysers often have automated cleaning and calibration routines. ISE sensors usually work with membranes which are prone to fouling and hard to clean automatically unless part of multi-parameter systems with dedicated wiping routines. Monitoring of sensor data in near real-time and automated anomaly detection can help to optimise the maintenance timing and efforts. Conventional lab measurements at sensor sites are important to validate and correct sensor data. An important consideration in maintenance and data quality of all high-frequency water quality sensor systems is the need for trained technical personnel. A common misconception is that automated water quality sensing equipment is ‘plug-and-play’, and this can lead to poorly resolved equipment performance, data quality, and data quantity.

## Data processing

As a final step in the decision workflow (Fig. 2), we highlight the need for adequate data processing. This section first describes different common types of anomalies in raw water quality sensor data and then evaluates recent advances in automated procedures for quality control and data cleaning. Automated data processing is needed as large sensor networks generate large amounts of data making manual data processing too time consuming. In addition, real-time anomaly detection routines can provide alerts to technicians for required maintenance. Real-time data processing can also improve live online data visualisations, such as in the Australian 1622WQ app (Vilas et al., 2020). Finally, standardised water quality sensor data optimisation routines can deliver more reliable data for process interpretation, modeling, and decision making.

### Types of anomalies in water quality sensor data

**Fig. 4:**
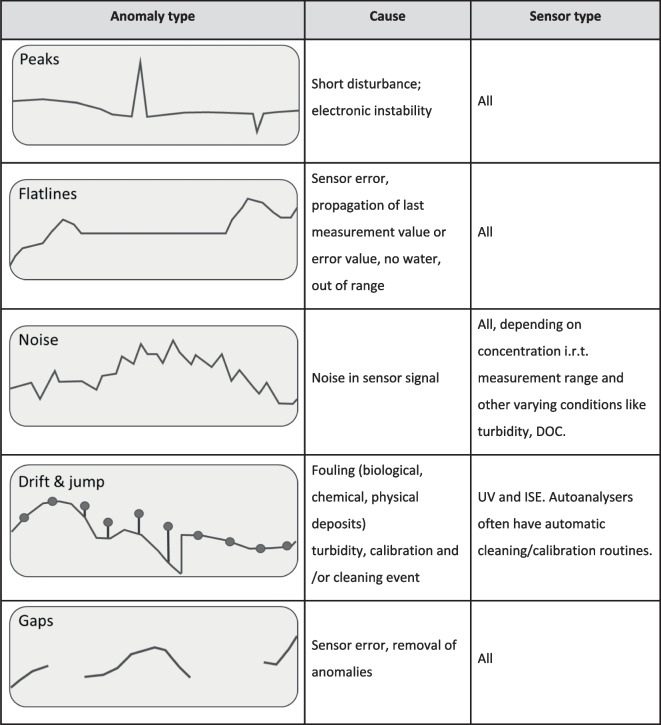
Common types of anomalies in water quality sensor data

Data validation and correction of high-frequency water quality data is highly complex because erratic measurements are hard to distinguish from real concentration variability. The highly variable and hard to predict solute concentrations in water make high-frequency water quality data validation much more complex as compared to other types of hydrological data (groundwater levels, surface water discharge). Common anomalies, together with their usual causes and their typical sensor types, are summarised in Fig. 4. Peaks (or lows) occur in UV and ISE sensors because of short disturbances or electronic instabilities. Auto-analysers are less sensitive to these disturbances because of the more controlled conditions and the pre-treatment of the samples. Flatlines are observed because of sensor or data transfer malfunctions. Noise is most typical when the measured concentrations are low in relation to the range of the sensor and can be caused by sensor instabilities and/or varying disturbances such as turbidity or DOC concentrations.

Drift in sensor data is usually caused by fouling for which ISE are especially sensitive. Wipers or air pressure cleaners reduce fouling in most optical sensors but are not effective on ISE membranes. Drift can also occur in UV sensors due to aging of the lamps, which can be minimised by periodic recalibration. After a period of drift, cleaning and calibration events often produce jumps in the sensor time series. As a last feature in Fig. 4, data gaps are common in water quality sensor time series due to sensor or data transfer failure or after removing anomalous data.

To be able to check for potential offsets, most high-frequency water quality stations are also still conventionally sampled and analysed in a laboratory. This is both useful for validation of the sensor data and for the monitoring of a broader range of water quality parameters. In most cases, the strength of conventional snapshot sampling lies in the accuracy of the measured values, while the strength of sensor data lies in capturing the temporal dynamics. Combining these strengths would produce optimal time series but, beyond use in validation studies (e.g. Jordan & Cassidy, 2011), no procedures or examples for this are reported yet in water quality literature.

### Available data processing tools

Techniques for automatic anomaly detection, smoothing, and noise correction are widely available, but are not yet widely applied to water quality data. High-frequency water quality monitoring has long been applied at a limited scale and data processing has typically been (and often still is) done manually by individuals who do not report their procedures. In this section, we provide an overview of published and openly available data processing procedures and routines applied for optimising high-frequency water quality datasets. To keep this overview concise, we have excluded data processing applications for other types of high-frequency data (e.g. hydrological data) and for low-frequency water quality data.

A limited number of research groups has recently published their water quality sensor data processing procedures and routines (Hawkins, 2021; Jones et al., 2022; Schmidt et al., 2023; Talagala et al., 2019; Veen et al., 2024; Zhang & Thorburn, 2022). The National Ecological Observatory Network also publishes their algorithms used for automated detection of anomalies including range, steps, spikes, and gaps (Sturtevant et al., 2022). For each of these groups, this effort was motivated by the wider scale use of water quality sensors which made visual data inspections and manual corrections too laborious and too hard to reproduce. These data processing procedures were also made public to share the knowledge and to aid transparency towards stakeholders.

Hawkins (2021), Jones et al. (2022), and Schmidt et al. (2023) all report a common order of data processing starting with (1) basic data preprocessing (e.g. time step harmonisation, data format checks), followed by (2) straight forward quality control (e.g. missing, impossible, out-of-range values, flatlines), (3) more complex quality control (anomaly detection, drift detection), and finally (4) gap filling routines.

A sophisticated framework for detecting outliers in water quality sensor data was published by Talagala et al. (2019) and is implemented in the R-package *oddwater*. This method focuses on abrupt changes in value, including sudden spikes, isolated drops, and level shifts within the time series. After basic preprocessing steps, eight unsupervised outlier scoring techniques were combined. This approach was also made available in the comprehensive python tool SaQC (System for automated Quality Control) published by Schmidt et al. (2023). SaQC also includes other multivariate outlier detection options, based on k-nearest neighbours (kNNs) and the STRAY (Search and TRace AnomalY) algorithms. Another promising anomaly detection method based on a wavelet-ANN (artificial neural network) model was applied to high-frequency river nutrient concentration data by Shi et al. (2018).

The Python package Pyhydroqc (Jones et al., 2022) also provides anomaly detection options for sensor data, including anomaly detection using dynamic thresholds or one step ahead predictions using long short-term memory (LSTM) and Autoregressive integrated moving average (ARIMA) algorithms. Pyhydroqc (Jones et al., 2022) also includes functionality for linear drift correction, while SaQC (Schmidt et al., 2023) has options for correction of both linear and exponential drift. These tools do not incorporate conventional snapshot sample data for corrections of drift or off-set.

Several options exist for filling in data gaps in water quality sensor time series. Pyhydroqc (Jones et al., 2022) for example uses the LSTM and ARIMA algorithms for making a forecast and backcast of the missing data, which are then cross-faded into a combined prediction. An overview of other univariate missing data imputation techniques applied to water quality sensor data is published by Zhang and Thorburn (2022). In their comparison study, SSIM (sequence-to-sequence imputation model, Zhang et al. (2019)) and Dual SSIM (Zhang & Thorburn, 2021) performed best for filling in relatively short (six datapoints) gaps.

When longer data gaps occur, multivariate gap filling procedures can be more efficient, valorising time series of simultaneously measured parameters like discharge, precipitation, electrical conductivity (EC), and turbidity. Examples of multivariate options for gap filling are LLS impute and PCA impute (Curceac et al., 2021). LSS impute first selects the best predictor variables based on Pearson, Spearman, and Kendall correlation coefficients. Subsequently, the missing values are predicted based on regression with these predictors. PCA impute also uses relations between multiple variables and is based on principal component analysis (PCA). The application of PCA models to impute missing values is also implemented in the missMDA R package (Josse & Husson, 2016). Barcala et al. (2023) explored several machine learning options for multivariate gap filling and found that random forest algorithms (Breiman, 2001) performed best in filling in relatively long (several weeks) data gaps in nitrate and TP time series. Random forest as well as Markov switching auto-regressive models (Spezia et al., 2021, 2023) are considered promising techniques for high-resolution water quality data processing.

### Data storage

Reliable procedures for data storage are crucial in water quality sensor applications. For purposes of transparency and reproducibility, raw data should always be stored, although some data optimisation already takes place within sensor software. The Nitratax UV NO_3_ sensor (Hach), for example, combines three measurements into a single output to reduce scatter. Raw data can be flagged in the database using quality control codes indicating the reason for flagging and/or the level of reliability. When optimised data series are produced, these can be stored separately. In practice, this is often done in steps, for example producing bronze (raw data), silver (basic optimisation), and gold (maximally optimised) data layers.

Storage of water quality sensor data should align with the FAIR guiding principles for data management, meeting principles of findability, accessibility, interoperability, and reusability (Wilkinson et al., 2016). Several national and international data storage standards exist for water quality measurements, although adjustments are usually needed to allow for storage of water quality sensor data.

### Data interpretation and application

After cleaning (and storage), the monitoring data are ready for further interpretation and application which often involves further processing steps such as visualisations, statistical analyses, modelling, and decision support systems. Here, the monitoring cycle loops back to the original monitoring objectives (MacDonald, 1994; Timmerman et al., 2000). That is to say asking if the collected data are sufficient for answering the original research questions? If not, the monitoring strategy needs reconsideration.

Data interpretation and application procedures are highly case specific and depend on the hydrochemical complexity of the research site and the objectives of the high-frequency monitoring application. Rode et al. (2016a) and Bieroza et al. (2023) provided examples of sensor data interpretation and applications with a focus on process understanding. In many cases, meteorological data and discharge data are used. For example, concentration-discharge relationships for storm events based on high-frequency data can help understanding solute transport processes (e.g. Lloyd et al., 2016).

## Conclusions

This paper was motivated by the desire to allow those new to the use of water quality sensors to benefit from the current international best practices in sensor applications, troubleshooting, and data processing. The deployment of high-frequency water quality monitoring sensor/analyser equipment is increasing and is expected to increase further in the future as regulatory monitoring becomes more important and climate change is expected to emphasise the relevance of extreme events for water quality. As outlined in this paper, water quality sensors have added value both for understanding pollutant transport and biochemical turnover processes and for operational water quality management such as compliance testing, load estimates, early warning, mitigation effect evaluation, and stakeholder engagement. However, current limitations of high-resolution water quality monitoring technology include the limited number of parameters, the vulnerability of the equipment for harsh outdoor conditions, and the costs for purchase, installation, and maintenance. The lack of international standardisation (NEN, ISO) and the challenges of handling and cleaning large volume of data captured from sensor networks may also hamper the uptake of high-frequency monitoring by water managers. Future work should focus on the development of low-cost, robust sensors to extend the range of water quality parameters, together with software that can facilitate the processing and interpretation of large data sets. Consensus and international standardisation on how to apply high-frequency water quality monitoring technology can also accelerate an efficient uptake in water quality management and is proposed as a priority.

## Data Availability

No datasets were generated or analysed during the current study.
